# Successful Vaginal Natural Orifice Transluminal Endoscopic Surgery (V-NOTES) Hysterectomy Under Spinal Anesthesia for an Obese Patient

**DOI:** 10.7759/cureus.97149

**Published:** 2025-11-18

**Authors:** Fatima Ba Khamis, Hala Bashir, Huda Manea, Haroutyoun Margossian

**Affiliations:** 1 Obstetrics and Gynecology, Dubai Health, Dubai, ARE; 2 Urogynecology, Dubai Health, Dubai, ARE

**Keywords:** hysterectomy, minimally invasive surgery, obesity, spinal anesthesia, urogynecology, vaginally assisted natural orifice transluminal endoscopic surgery (v-notes)

## Abstract

Vaginal natural orifice transluminal endoscopic surgery (V-NOTES) hysterectomy combines the scarless vaginal hystrectomy approach with the precision of laparoscopy, offering less postoperative pain, quicker recovery, and fewer complications compared to abdominal or conventional laparoscopic hysterectomy. While general anesthesia is commonly used for laparoscopic procedures, spinal anesthesia offers benefits that are especially valuable in obese patients. We report the case of a 46-year-old woman (body mass index: 30 kg/m²) with stage 2 utero-vaginal prolapse and rectocele who underwent V-NOTES hysterectomy with anterior-posterior vaginal repair and high intraperitoneal colpopexy under spinal anesthesia. The patient received intrathecal bupivacaine with morphine, followed by sedation with midazolam. The procedure lasted for three hours with low CO₂ insufflation pressure (8-12 mmHg) and minimal Trendelenburg positioning <10 degrees. She remained stable intraoperatively, with an estimated blood loss of 400 cc. Postoperatively, she experienced minimal pain controlled with oral analgesics, no nausea or vomiting, and was ambulatory within hours. To our knowledge, this is the first reported case of V-NOTES hysterectomy done under spinal anesthesia in an obese patient, highlighting its feasibility, safety, and potential for broader clinical use.

## Introduction

Hysterectomy is the surgical removal of the uterus, and it can be performed through different surgical approaches such as abdominal, laparoscopic, and vaginal. Vaginal natural orifice transluminal endoscopic surgery (V-NOTES) hysterectomy was first reported in 2012 [1,2]. It combines the transvaginal access of vaginal hysterectomy with the visualization and precision of laparoscopy, without requiring abdominal incision. Compared to abdominal and conventional laparoscopic hysterectomy, V-NOTES hysterectomy is less invasive, offers faster recovery, decreases postoperative pain, reduces wound complication rates, decreases incisional hernias, and has no visible incisions [3]. General anesthesia (GA) has been the preferred choice for laparoscopic surgeries, as it allows for controlled ventilation, making pneumoperitoneum insufflation and positioning the patient in the Trendelenburg position [3] safer and more manageable. Although there are a few reports of safe administration of spinal anesthesia (SA) with laparoscopic surgery, it is less widely accepted compared to GA [4,5]. SA offers various advantages, including less postoperative pain [3], allowing for earlier mobilization and faster return to normal activities, thus improving the recovery period [3]. Furthermore, GA requires airway management that may pose additional risks for respiratory distress and airway aspiration compared to SA, especially in obese individuals. The postoperative period can be complicated more frequently by nausea and vomiting [3].

This case report describes the effective use of V-NOTES hysterectomy under SA, focusing on its practicality, patient results, and its advantages. The procedure was performed on a 46-year-old woman with a body mass index (BMI) of 30 kg/m², who had stage 2 utero-vaginal prolapse. To our knowledge, this is the first case of V-NOTES hysterectomy performed under SA for an obese patient. This case report aims to contribute to the knowledge on this approach to allow for the development of guidelines and standard care for the patients.

## Case presentation

A 46-year-old woman, para 1+2, with a previous one full-term uncomplicated vaginal delivery. She had a history of a first-trimester miscarriage and right salpingectomy for ectopic pregnancy. She was referred to the urogynecology clinic at Latifa Hospital for evaluation of urge urinary incontinence and chronic constipation.

**Figure 1:**
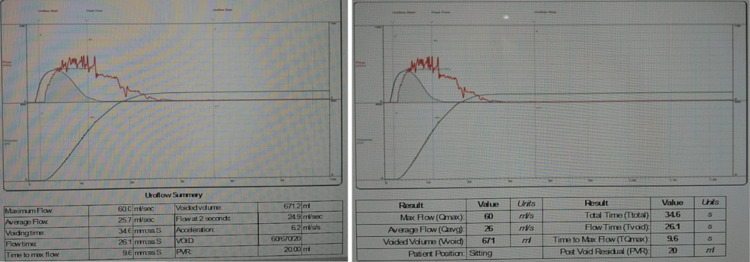
Uroflowmetry findings.

**Figure 2:**
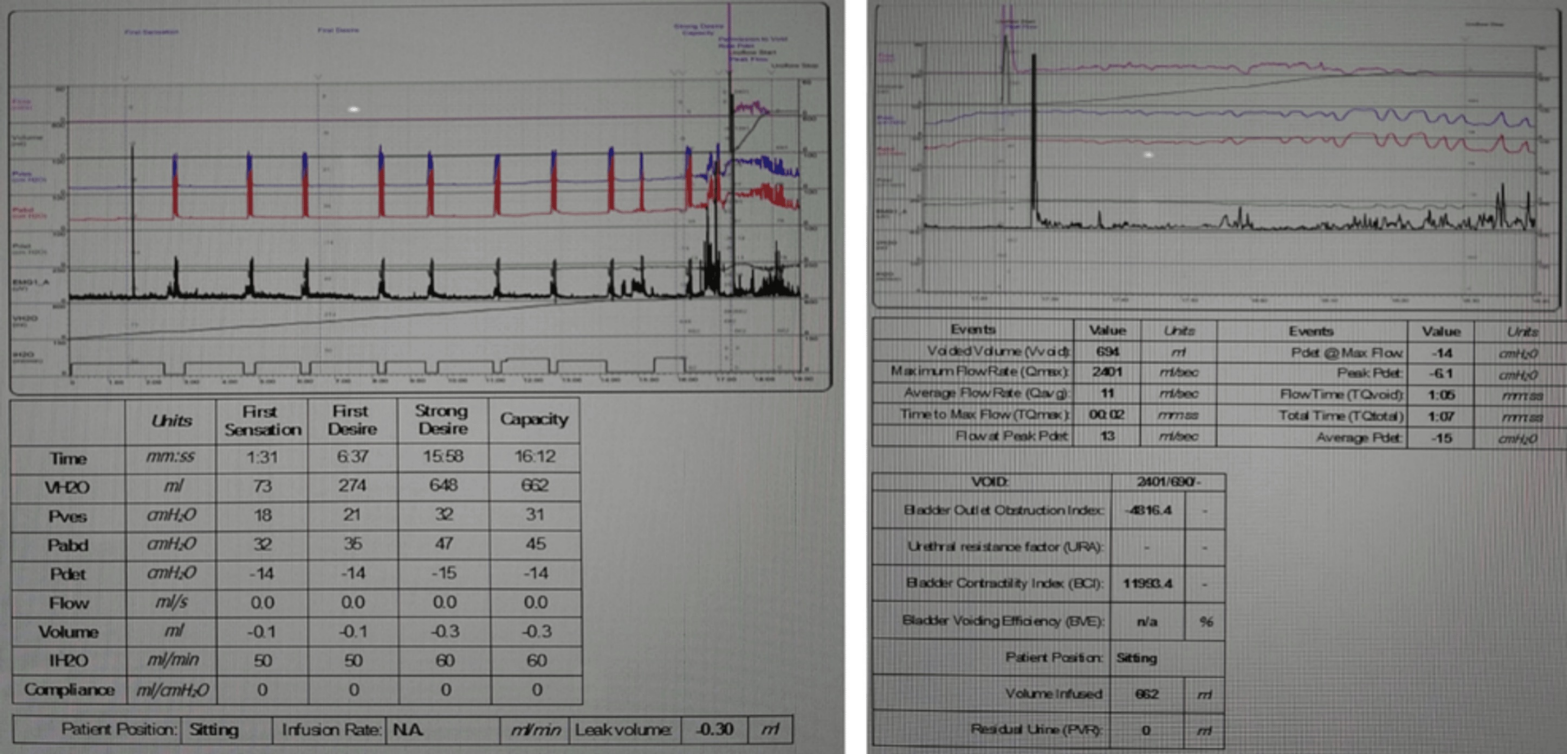
Urodynamic study showing filling phase on the right and voiding phase on the left.

**Figure 3:**
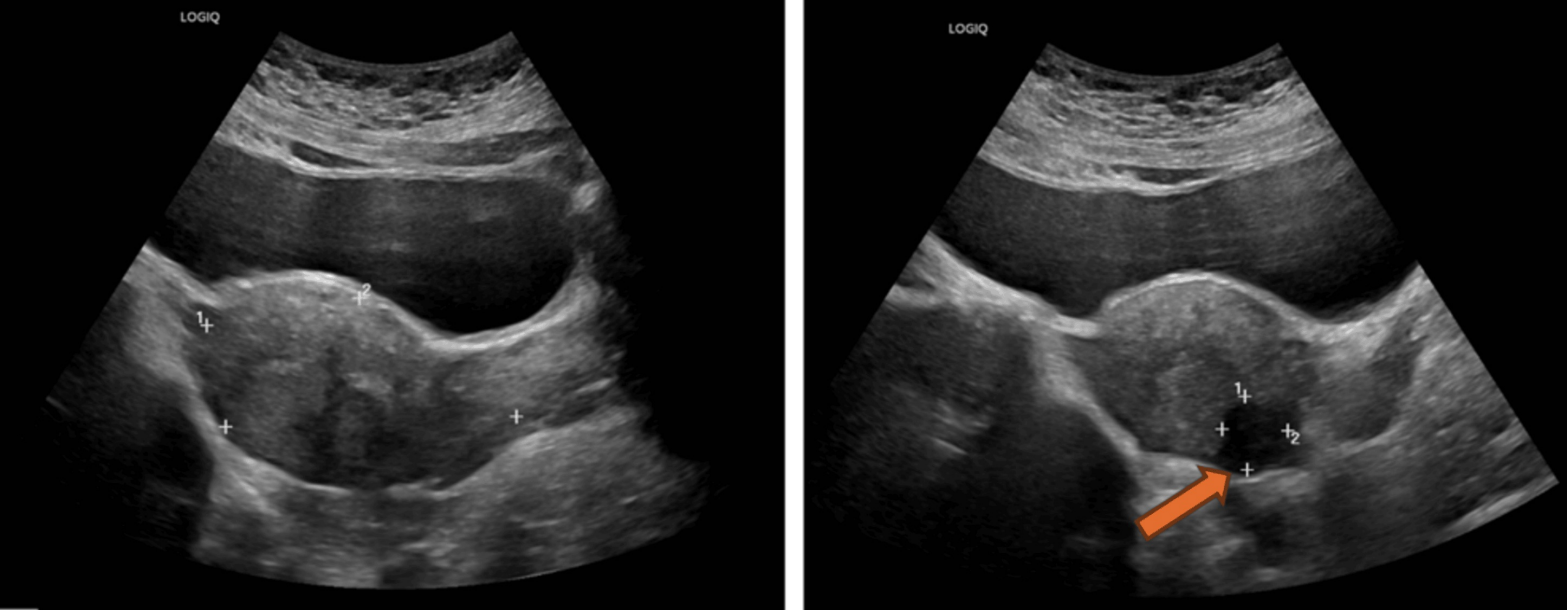
Pelvic ultrasound showing a small posterior wall intramural uterine fibroid measuring 1.9 × 1.7 cm.

**Figure 4:**
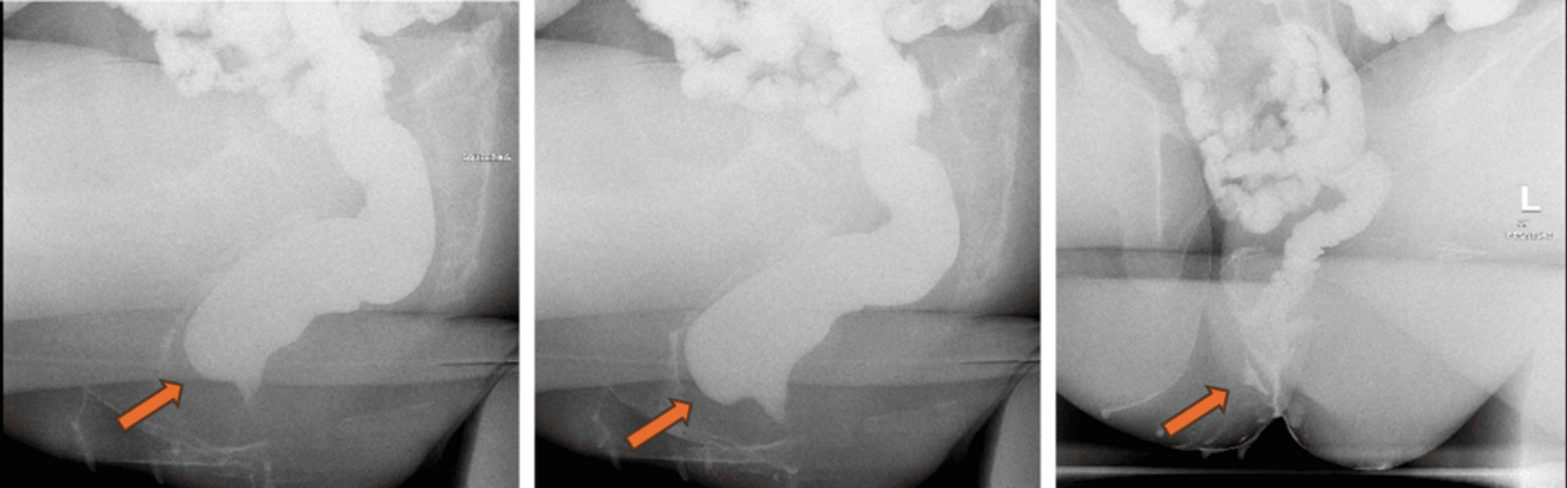
Defecography showing pelvic floor prolapse, intra-anal mucosal intussusception, and anterior rectocele.

Physical examination revealed a BMI of 30 kg/m², stage 2 uterine prolapse, and stage 2 rectocele. A urodynamic test showed no detrusor overactivity and no stress urinary incontinence (Figures 1, 2). A pelvic ultrasound was within normal, aside from a small intramural fibroid (Figure 3). A defecography study revealed pelvic floor prolapse, intra-anal mucosal intussusception, and anterior rectocele (Figure 4). She was treated initially with conservative management, but her symptoms persisted, impacting her quality of life; hence, she was deemed a suitable candidate for V-NOTES hysterectomy, with anterior and posterior vaginal repair and high intraperitoneal colpopexy using uterosacral ligaments.

**Figure 5:**
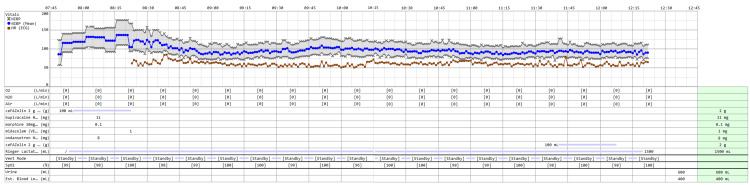
Intraoperative anesthesia record.

 Preoperative anesthesia assessment classified her as class 2 according to the American Society of Anesthesiologists classification [6]; the patient expressed a preference for SA. Intraoperatively, the patient was administered SA with bupivacaine 11 mg mixed with 0.1 mg morphine to prolong postoperative analgesia. After 15 minutes, the patient was sedated with midazolam 1 mg. The surgical procedure lasted approximately three hours. The procedure was done under low intraperitoneal CO₂ insufflation pressure (8-12 mmHg) and a minimal 10-degree Trendelenburg position. The patient remained vitally stable throughout the surgery (Figure 5), with a mean arterial pressure above 65 mmHg and a total blood loss of 400 cc.

The patient experienced minimal postoperative pain and was able to ambulate within hours after the surgery. She did not have any nausea or vomiting, voided normally after the removal of the urinary catheter, and regained normal bowel function by the second postoperative day. She was discharged on the second postoperative day with oral analgesics.

The patient was seen three weeks after her surgery for a follow-up. She reported significant improvement in her symptoms, with resolution of urinary urge incontinence symptoms and constipation. Her physical examination revealed normal healing of the vaginal incision.

## Discussion

Introduced in 2012 [1,2], V-NOTES hysterectomy is noted for its safety and effectiveness, potentially offering better visibility and access during surgery compared to the traditional vaginal hysterectomy method [2] in certain selected cases [7], as it combines both scarless vaginal approach and laparoscopic precision and visibility.

GA is frequently preferred in laparoscopic procedures because of the impact these surgeries have on the respiratory and cardiovascular systems. It also provides better bowel relaxation and a lower risk of hypercapnia caused by CO₂ accumulation during pneumoperitoneum [7]. However, few reports studied the combination of V-NOTES surgery with SA, showing that it can be administered safely, without compromising patient comfort or the surgical technique [3,7,8].

Intra-abdominal insufflation, along with the Trendelenburg position, used to visualize the pelvic organs better in V-NOTE surgeries, can compromise the respiratory function by elevating the diaphragm, raising the intrathoracic pressure, and decreasing the lung compliance [9,10]. Moreover, the increase in intra-abdominal pressure in laparoscopic surgeries affects cardiac function by decreasing blood return from the inferior vena cava to the heart. Hence, it is recommended to maintain the intra-abdominal pressure between 8 and 12 mmHg and the Trendelenburg position below 20 degrees during the operation [3].

According to the World Health Organization (WHO), a BMI of 30 kg/m² or higher classifies an adult as obese [11]. Obesity is significant in determining the most appropriate and safe anesthetic approach, especially when comparing SA and GA. In patients with higher BMI, GA carries increased risks such as difficult intubation, reduced functional residual capacity, and a higher chance of pulmonary aspiration, atelectasis, and other respiratory complications [12].

In our case, the patient had a BMI of 30 kg/m², placing her in Obesity Class I according to the WHO classification [11]. To our knowledge, there have been three publications about V-NOTE surgery done under SA, but this is the first case in an obese individual [3,7,8]. Typically, patients post-V-NOTE surgeries experience lower pain [8] due to lower insufflation pressure used in the procedure, making the possibility of same-day discharge feasible [13]. Our patient experienced a smooth postoperative period with minimal pain controlled with simple oral analgesia; she had no nausea and vomiting (PONV) post-anesthesia, aiding early mobilization and promoting quicker recovery time and fast return to normal bowel function. There are reports of minimal risk of shoulder tip pain post-SA, which may occasionally necessitate conversion to GA [3,7]; however, our patient had no complaints.

This case demonstrates the effective application of V-NOTES hysterectomy with SA. It is important to note that specialized training and equipment are required for v-NOTES hysterectomy. Surgeons need to be skilled in endoscopic and urogynecology procedures. The surgery must be performed in a timely manner under SA to achieve the best results [3].

## Conclusions

As demonstrated in our case, V-NOTES hysterectomy performed under SA can be a feasible and successful approach, offering a smoother postoperative course with minimal pain, rapid recovery, and early mobilization. This technique may represent a valuable alternative for selected patients, particularly those in whom GA poses higher risks, such as patients with obesity. However, broader clinical experience and larger studies are essential to further validate its safety, reproducibility, and potential to become an established standard in minimally invasive gynecologic surgery.
